# How Lateral Connections and Spiking Dynamics May Separate Multiple Objects Moving Together

**DOI:** 10.1371/journal.pone.0069952

**Published:** 2013-08-02

**Authors:** Benjamin D. Evans, Simon M. Stringer

**Affiliations:** Oxford Centre for Theoretical Neuroscience and Artificial Intelligence, Department of Experimental Psychology, University of Oxford, Oxford, United Kingdom; University of Texas at San Antonio, United States of America

## Abstract

Over successive stages, the ventral visual system of the primate brain develops neurons that respond selectively to particular objects or faces with translation, size and view invariance. The powerful neural representations found in Inferotemporal cortex form a remarkably rapid and robust basis for object recognition which belies the difficulties faced by the system when learning in natural visual environments. A central issue in understanding the process of biological object recognition is how these neurons learn to form *separate* representations of objects from complex visual scenes composed of *multiple* objects. We show how a one-layer competitive network comprised of ‘spiking’ neurons is able to learn separate transformation-invariant representations (exemplified by one-dimensional translations) of visual objects that are always seen together moving in lock-step, but separated in space. This is achieved by combining ‘Mexican hat’ functional lateral connectivity with cell firing-rate adaptation to temporally segment input representations of competing stimuli through anti-phase oscillations (perceptual cycles). These spiking dynamics are quickly and reliably generated, enabling selective modification of the feed-forward connections to neurons in the next layer through Spike-Time-Dependent Plasticity (STDP), resulting in separate translation-invariant representations of each stimulus. Variations in key properties of the model are investigated with respect to the network’s ability to develop appropriate input representations and subsequently output representations through STDP. Contrary to earlier rate-coded models of this learning process, this work shows how spiking neural networks may learn about more than one stimulus together without suffering from the ‘superposition catastrophe’. We take these results to suggest that spiking dynamics are key to understanding biological visual object recognition.

## Introduction

In the primate visual system, increasingly complex representations are developed at successively higher layers in the ventral stream hierarchy [1], [2] until individual neurons respond selectively to particular faces [3] or objects [4]. In a way that still eludes many artificial systems, these neurons also respond invariantly to a range of transformations of their preferred stimuli including translations [5]–[7], changes in size [7], [8] and view [9], [10]. Models which have sought to understand the formation of such transformation-invariant representations in Inferotemporal cortex (IT) have largely used training paradigms where stimuli are presented individually. An important question concerning this learning process therefore remains – how can the visual system become selective for *individual* objects (or faces) when it only experiences natural scenes composed of *multiple* objects?

Previous attempts to simulate this process with rate-coded models of the visual system have encountered difficulties whereby the coactivity of neurons representing features of each stimulus leads to false conjunctions between features belonging to different stimuli – the ‘superposition catastrophe’ [11]. This problem is further exacerbated by rate-based Hebbian learning, whereby the stimuli are associated onto the same (simultaneously coactive) output neurons leading to combined representations after learning. The consequence of this learning is that the same response is evoked by presenting any of the individual stimuli, thus undermining the discriminability of the model. In order to avoid this problem, rate-coded neural networks are commonly trained by presenting only one stimulus at a time to ensure that only features from one particular stimulus are associated onto an output neuron [12]–[14], but leaving the training paradigm lacking in ecological validity. However, recent research has uncovered a number of mechanisms for overcoming this problem.

VisNet, a model of the ventral visual stream consisting of a hierarchical, feed-forward series of rate-coded competitive networks [12] was presented with multiple stimuli transforming (shifting or rotating) in different combinations. It was found that if the pool of stimuli was large enough and a sufficient number combinations was presented during the learning phase, the statistical decoupling between the objects forced the competitive networks to form independent representations of the stimuli in the output layer [15], [16]. However, achieving this required an extensive training regime where objects were repeatedly seen in different combinations, leaving the problem of how objects may be disentangled even when they are *always* (or very often) seen together.

Another mechanism discovered in VisNet solving the ‘superposition catastrophe’ of multiple object presentations was found to depend upon independent movement of the stimuli [17]. Although there were only two stimuli in each experiment (negating the possibility of statistical decoupling by showing different combinations of stimuli), presenting the objects rotating at different speeds allowed the competitive networks to similarly form transformation-invariant *separate* representations in the network’s output layer. While independence of movement is typically a reasonable assumption to make of objects in a natural scene, it may not always be valid, (for example when neither the objects nor the viewer moves, such as when viewing a photograph). This leaves the possibility that simple spatial separation of objects may be sufficient to learn independent representations of them.

Traditionally, visual perceptions have been assumed to be represented by the activation level or firing-rate of neurons, known as the *spike-count hypothesis*. Indeed, previous work has suggested that the average firing rate over a short window, 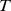, from the onset of the stimulus, 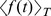, is the relevant code for transmitting information [18] with estimates for 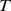 of 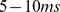 [19], 20, or even up to 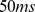 [20]. While the majority of the information of output neurons’ responses may be contained within their firing rates [18], the timing of their action potentials may still be important for how networks self-organize during learning, potentially allowing them to overcome the limitations inherent in a simpler rate-coded counterpart.

In one spiking neural network, the ‘binding problem’ of separating stimuli within large receptive fields was overcome through an attentional mechanism [21]. This was implemented by selectively reducing the firing threshold of particular neurons throughout the layers, whose receptive fields fell in the attended region. However, while attention may play a role in some circumstances, there must still be an automatic mechanism for unattended scene segmentation.

Previous work with a network of spiking neural networks has found that, under the right conditions, competing populations of neurons will tend to push one another out of phase and thereby alternate their respective perceptual representations through time in a phenomena dubbed ‘perceptual cycles’ [22]. This mechanism has been demonstrated to allow for both segmentation and binding (feature linking) of a visual scene [23] and may be used by spiking neurons to overcome the difficulties presented by multi-object training paradigms.

Once such an anti-phase dynamic is established in the inputs, it is hypothesised that postsynaptic excitatory cells in subsequent layers will be able to learn (through Spike-Time-Dependent Plasticity in the feed-forward synapses) about each object independently of the others as they translate across the input layer. Hence, without independent motion or the extensive training of statistical decoupling, the binding and segmentation occurring naturally through the inputs’ temporal code should allow transformation-invariant cells for each object to rapidly and naturally form in the output layer. This would be in line with previous speculation that automatic scene segmentation may increase the learning ability in downstream areas of the brain [22]. It would also suggest that spiking neurons may be a more appropriate level of abstraction on which to capture the learning processes in biological object recognition [24].

In some of our earlier work, we demonstrated how a more biophysically accurate spiking neural network, (explicitly modelling individual action potentials rather than a time-windowed average of activity) could form transformation-invariant representations of objects presented individually during training [25]. In this paper it is demonstrated how such a spiking neural network can utilise the richer spiking dynamics to learn separate translation-invariant representations of visual stimuli which are always seen together and always moving together, but spatially separated in the visual field. This is achieved by combining a ‘Mexican hat’ network architecture with adapting spiking neurons learning through a spike-time-dependent learning rule. There follows an introduction to the key components required for the operation of this model in more detail and a summary of how they interact to achieve separate translation-invariant representations.

### Conditions for Synchronous Cell Assemblies

Previous work has revealed several key features required of a model to form synchronous assemblies of neurons representing a particular stimulus (‘feature linking’) and to generate an anti-phase relationship between competing (input) representations. One such requirement is for (short-range) lateral excitatory connections between principal excitatory cells. These form a mutually supportive basis for synchronising the spike volleys of spatially proximal features of a particular object, while inhibitory interneurons tend to desynchronise representations of different objects. The second requirement is either conduction delays [26], varying postsynaptic potential decay rate [27] or cell firing-rate adaptation [22], [23], [28], [29]. Together, these features act to generate periodic firing in each population of principal cells.

The conditions for synchronisation and desynchronisation were studied in detail for pairs of neurons with conduction delays [26]. In general, four regimes were identified in their analysis of a simple two neuron system with excitatory or inhibitory coupling, and then confirmed with larger scale simulations. These regimes are as follows: (1) mutual excitatory connections without delays cause synchrony (quickly); (2) mutual excitatory connections with delays cause desynchrony; (3) mutual inhibitory connections without delays cause desynchrony; and (4) mutual inhibitory connections with delays cause synchrony (slowly). Similarly, 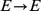 synapses with fast PSP decay and 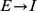 synapses with slow PSP decay lead to synchrony, whilst the opposite combinations lead to desynchrony [27].

#### Delayed self-inhibition

In the work presented here, we chose cell firing-rate adaptation as the mechanism by which periodic firing is generated through a ‘time-delayed neuronal self-inhibition mechanism’ [30] as this is a common feature of many spiking neuron models and found throughout the brain. When calcium ions (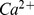) enter the cell through voltage-gated L-type channels during an action potential [31], Calcium-gated Potassium (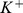) channels are opened. The resultant flow of Potassium ions across the cell membrane is known as the after-hyperpolarization current (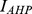). This makes the membrane more ‘leaky’, and so has a shunting effect upon the cell membrane potential, making it harder to reach spiking threshold again for a time course governed by the decay rate of 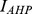 as it exponentially returns to 0 [30], [32].

Alternately, this process has been modelled with a ‘dynamic threshold’ but contrary to the experimental evidence [33] the time constant of adaptation in the dynamic threshold model decreased as a function of the input current intensity [30] and so the current subtraction model is used here. Interestingly, when operating in conjunction with Spike-Time-Dependent Plasticity (STDP), adaptation has been found to yield almost optimal information transmission [34]. It was found in the simulations described below that this mechanism facilitated the emergent behaviour of interest in a homogeneous population of principal cells.

#### Lateral interactions

Lateral connections are commonly found throughout the visual cortex [35] and according to the analysis of Nischwitz and Glunder, [26], are another key property for generating the firing dynamics of interest. Rather than using axonal conduction delays as in previous work [26], a ‘Mexican hat’ profile is used to mediate interactions between neurons within a layer. With this connectivity, features spatially close to one another in a visual projection will provide mutually supportive excitation leading to synchronous firing. Since such neighbouring neurons are likely to represent features of the same stimulus, the appropriate neurons will therefore be bound into a coherent stimulus percept through synchrony. Conversely, longer-range inhibition should desynchronise neural populations with respect to those representing other simultaneously presented stimuli elsewhere in the visual field. Such features of lateral connectivity therefore allow the temporal binding of the proximal features of one stimulus, in anti-phase oscillations to features of another (more distant) object.

Synchronising feature representations which belong to the same stimulus (in this case on the basis of spatial proximity) to produce a coherent percept of the stimulus is known as the ‘binding-by-synchrony’ hypothesis [36], [37]. Importantly, this mechanism elegantly avoids the combinatorial explosion of cells which would otherwise be required in a system where conjunctions of features are represented explicitly. This idea is supported by neurophysiological recordings showing the synchronised oscillations of visual neurons with similar orientation preferences when presented with a common input [38] and psychophysics studies showing that stimuli are harder to differentiate when presented synchronously [39], suggesting synchronous oscillations underpin the conscious perception of a stimulus.

### Overview of Model Dynamics

Here it is demonstrated how the Gaussian profile of excitatory lateral connections helps to synchronise the discharges of local clusters of neurons in the input layer which represent an individual visual object, while the long-range (global) inhibitory connections desynchronise the action potentials between spatially separate clusters of input neurons (which represent different visual objects). The two visual input representations are thus pushed out of phase with respect to each other in this manner. Furthermore, the cell firing-rate adaptation ensures that one representation does not continually suppress the other but that the volleys of spikes oscillate between the different stimuli on a time-scale of roughly 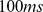.

With the dynamics of the input layer representations settling into the described anti-phase oscillations, the strength of the plastic feed-forward excitatory connections projecting to the next layer are selectively modified through STDP. Specifically, there is long-term potentiation (LTP) if the presynaptic spikes occur in the order of 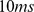 before the postsynaptic spikes and long-term depression (LTD) if this order is reversed [40]. If separate output neurons fire between the oscillations of the two input representations, they will experience LTP for only one stimulus (and LTD for the other if the frequency of oscillation is sufficiently high). The effect of this is that separate pools of output neurons (determined by the initial random feed-forward connectivity) learn to respond selectively to only one of the synchronised input clusters representing a particular stimulus.

**Figure 1:**
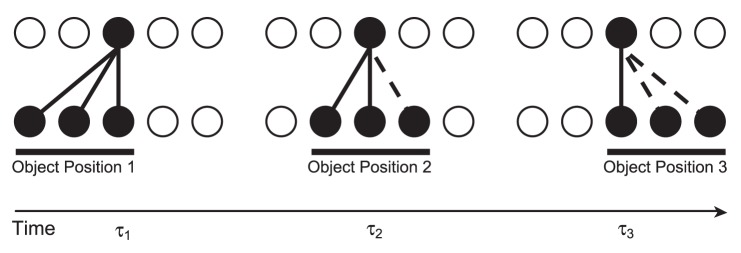
Transformation-invariance with the CT learning mechanism. In the initial position at the first transform time (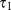) the input neurons randomly activate a set of postsynaptic neurons (due to the random synaptic weight initialisation) and the synaptic connections between the active input and output neurons will be strengthened through Hebbian learning. If the second transform at 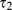 is similar enough to the first, the same postsynaptic neurons will be encouraged to fire by some of the same connections potentiated at 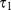 and the input neurons of the second transform will have their synapses potentiated onto the same set of output neurons. This process may continue (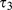) until there is very little or no resemblance between the current and the initial transforms. In addition to changes in retinal location, the same principles will apply to build other types of transformation-invariance. For example, changes in view and scale will be accommodated through the same process, provided that there is sufficient overlap of afferent neurons between the transforms.

This dynamic may be combined with the Continuous Transformation (CT) learning mechanism to achieve translation-invariant output representations. CT learning is a biologically plausible mechanism for guiding the development of transformation-invariant visual representations [14], [25]. Similar transforms of a stimulus are likely to activate many of the same upstream (afferent) neurons, thereby leading to the activation of, and association onto, the same set of downstream (efferent) neurons. This principle is most easily understood in the case of translation invariance (as detailed below and illustrated in Figure 1) where each transform represents a small change in retinal position. However, the same principle may be naturally extended to changes in viewing angle or scale provided that these transforms activate significantly overlapping sets of neurons. As such, the simulations provided here with translation invariance serve as examples of the more general case of forming *transformation*-invariant representations by virtue of the same mechanisms.

By continuously transforming (shifting) the stimuli on the input layer, the similarity between transforms belonging to each particular stimulus is high. Due to this similarity, the CT learning mechanism is able to build the desired output representations using spike-time-dependent learning in the feed-forward connections according to the following process. Presentation of an initial transform in the input layer will excite a set of postsynaptic neurons and through the Hebbian (STDP) learning rule, will strengthen the synapses between those cells. If there is enough overlap (similarity) between the original and the new transform, the same postsynaptic neurons will be excited, causing potentiation of the synapses from the input neurons of the current transform. This process can continue across a series of overlapping transforms until they are all mapped onto the same output cells.

In this paper, it is shown how these neural mechanisms may operate together during learning to produce stimulus-specific and translation-invariant output cells when the visual objects have always been presented moving together in lock-step during training but physically separated in space. This network behaviour relies upon the explicit modelling of the times of spikes, together with STDP in order to obtain the necessary dynamics which would not be possible in earlier *rate-coded* models such as VisNet [15]–[17].

## Methods

### Model Architecture

To investigate the role of spike-timing in the segmentation of multiple stimuli, a neural network was created consisting of conductance-based integrate-and-fire neurons, (gLIF), which model the individual action potentials. To test the utility of input segmentation for learning in downstream neurons, the model consisted of two layers of excitatory (principal) cells, fully connected with feed-forward plastic synapses [41], while all other synaptic weights were fixed. The principal cells also featured cell firing-rate adaptation to provide a mechanism of self-inhibition and each layer had a separate pool of inhibitory interneurons to provide competition between the principal cells of each layer.

Correlated firing of principal cells responding to the same object was encouraged by a fixed ‘Mexican hat’ connectivity profile in the input layer (with exponentially decreasing excitatory connection strength). Conversely, anti-correlated firing of (more distant) neurons representing different objects was encouraged by the uniform strength of connections between principal cells and inhibitory interneurons. The input layer contained 512 excitatory cells (arranged in one dimension) to provide enough room for multiple translating stimuli (while the output layer was a 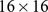 grid). For a summary of the network parameters used throughout the simulations, please refer to Table 1.

**Table 1:** Default network parameters.

Network Parameters	Symbol	Value
Cue current	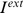	0.75 nA
Cue period {training, testing}	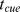	{500, 500} ms
Number of training epochs	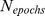	10
Time step for numerical integration	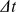	0.02 ms
Number of layers	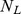	2
Number of excitatory cells per layer	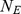	{512, 256}
Number of inhibitory cells per layer	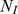	{128, 64}
Prob. of 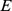 cell synapsing with afferent feed-forward 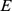 cell	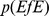	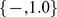
Prob. of 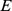 cell synapsing with afferent lateral 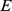 cell	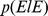	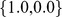
Prob. of 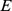 cell synapsing with afferent 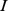 cell	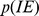	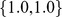
Prob. of 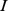 cell synapsing with afferent 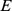 cell	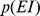	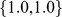
Prob. of 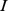 cell synapsing with afferent 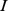 cell	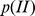	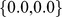
Standard deviation of lateral connection strength	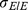	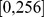

Sets of values are indicated by braces whereby the values correspond to the parameters used for each of the two layers of neurons (or the training/testing periods in the case of the cue period). Square brackets are used to indicate ranges of parameters which were explored through simulations.

### Neuron Model Description

The leaky integrate-and-fire neuron is principally defined by a differential equation describing the evolution of its cell membrane potential given by Equation 1, with accompanying parameters in Table 2. The synaptic currents perturbing the cell membrane potential are described by Equation 2 (and the accompanying parameters in Table 2) with the dynamics of the conductance-based synapses described by Equation 3 and the parameters of Table 3. This model also incorporates an adaptation current triggered by calcium-gated potassium channels [30] with a coupled equation to describe the Potassium channel dynamics given in Equation 4 (and accompanying parameters in Table 2). Both the cell membrane potential and adaptation current are also governed by the after-spike resetting conditions of Equation 5 (with the parameters also given in Table 2).
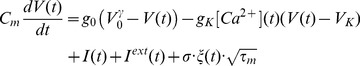
(1)


**Table 2:** Cellular parameters.

Cellular Parameters	Symbol	Value
Excitatory cell somatic capacitance	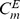	500 pF
Inhibitory cell somatic capacitance	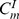	214 pF
Excitatory cell somatic leakage conductance	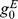	25 nS
Inhibitory cell somatic leakage conductance	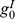	18 nS
Excitatory cell membrane time constant	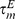	20 ms
Inhibitory cell membrane time constant	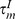	12 ms
Excitatory cell resting potential	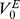	-74 mV
Inhibitory cell resting potential	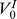	-82 mV
Excitatory firing threshold potential	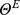	-53 mV
Inhibitory firing threshold potential	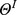	-53 mV
Excitatory after-spike hyperpolarization potential	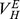	4 mV
Inhibitory after-spike hyperpolarization potential	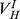	-58 mV
Excitatory reversal potential	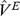	0 mV
Inhibitory reversal potential	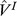	-70 mV
Absolute refractory period	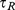	2 ms
Increase in adaptation (potassium) conductance	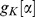	6 nS
Potassium reversal potential	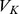	-80 mV
Adaptation (calcium decay) time constant	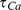	50 ms

The leaky integrate-and-fire parameters used by default throughout this paper were taken from Troyer et al. [43] (derived originally from cortical electrophysiological recordings [68]) with the adaptation parameters mostly taken from Liu & Wang [30].

**Table 3:** Synaptic parameters.

Synaptic Parameters	Symbol	Value		
Synaptic neurotransmitter concentration	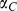	0.5		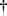
Proportion of unblocked NMDA receptors	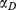	0.5		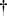
Presynaptic STDP time constant	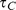	15	ms	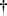
Postsynaptic STDP time constant	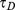	25	ms	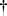
Synaptic learning rate	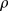	0.1		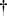
Plastic ( 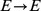 ) synaptic conductance range	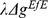	[0, 3.75]	nS	*
Lateral ( 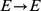 ) synaptic conductance range	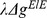	[0, 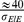 ]	nS	*
Change in synaptic conductance ( 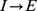 )	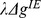	5.0	nS	*
Change in synaptic conductance ( 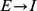 )	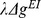	5.0	nS	*
Excitatory-Excitatory synaptic time constant	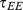	2	ms	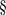
Inhibitory-Excitatory synaptic time constant	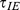	5	ms	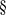
Excitatory-Inhibitory synaptic time constant	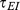	2	ms	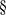

The synaptic conductance time constants were taken from the same studies as the cellular parameters [43], [68]) as indicated by 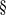. Plasticity parameters (denoted by 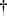) are taken from Perrinet et al. [41]. Parameters marked with * were tuned for the reported simulations and ranges were systematically explored where indicated by square brackets.

The time constant of the cell membrane (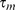) is broken into its component parts, the capacitance, 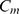 and leakage conductance, 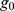 (inverse of the membrane resistance, 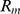), such that 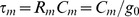. The membrane reversal potential (which the 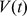 moves towards in the absence of stimulation) is symbolised by 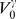, with 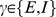 denoting the class of neuron, (either Excitatory or Inhibitory).

For biological realism, the cell membrane potential model (Equation 1) includes Gaussian noise of zero mean and standard deviation 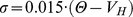 [42]. Here, 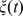 is a Wiener (Gaussian) variable (where 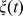 represents 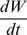) satisfying the definition of the Wiener process such that 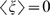 and 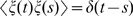, where 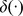 is the Dirac delta function. The noise amplitude is scaled by 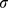, (since 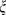 has unit variance) set to 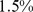 of the difference between the firing threshold 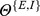 and the hyperpolarization potential 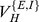.

The sum of synaptic currents flowing into the cell is represented by 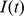 (described in Equation 2) while current from direct external stimulation is denoted by 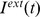.
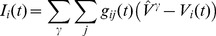
(2)


Here 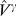 represents the reversal potential of a particular class of synapse (denoted again by 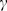) which consists of Excitatory and Inhibitory neurons, 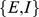 and 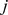 indexes the presynaptic neurons of each class. Activation of a particular synapse will therefore make the membrane more permeable to the species of ion determined by the synapse’s class 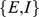 and will therefore drive the cell membrane potential more rapidly towards the reversal potential for that class (above or below the firing threshold respectively).

Equation 3 describes the dynamics governing the conductance of a particular synapse, 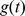. The conductance of each synapse (indexed by 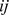) is governed by a decay term 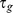, which varies according to the class of synapse, denoted 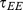, 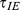 and 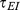, with corresponding parameters given in Table 3. A Dirac delta function describes the incoming presynaptic spikes, where 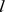 indexes over their arrival times at the synapse. This model neuron therefore neglects the shape of the action potential, as the present work is concerned only with their timing.
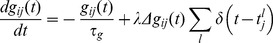
(3)


The synaptic efficacies for each class of synapse 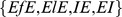 are modelled as a change in conductance at a particular synapse upon the arrival of an incoming spike. Hence, the efficacy (weight) is determined by the product of 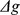, which is bounded in the range 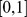 but set individually for all synapses and 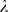, the biological scaling constant in 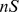, which is set individually for each synaptic class. The strengths of excitatory feed-forward synapses (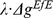) were plastic, modified by the STDP learning rule (described below) in the range 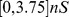. The strength of these synapses were lower than the default due to the simultaneous presentation of multiple stimuli. All other classes of synapse were fixed (non-plastic) as described below and detailed in Table 3.

To provide a mechanism of self-inhibition, an additional potassium-based (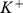) leakage conductance, 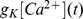, activates following recent spiking activity. This variable denotes the (potassium) conductance of the cell membrane, 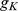, (resulting from the unblocking of potassium channels) due to a particular calcium concentration, 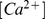. The resultant adaptation current (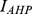) leaking out of the membrane drives the membrane potential towards the potassium reversal potential, 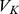, making it harder for the neuron to reach its firing threshold. The duration of this impeding effect is determined by the time course of the calcium concentration’s 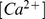 (and hence the adaptation current’s) decay back to 0 (Equation 4), characterised in this model by the time constant 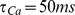 [30].
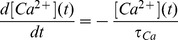
(4)


The auxiliary after-spike resetting is given by Equation 5. If the cell membrane potential reaches the cell’s firing threshold, 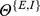, then the cell membrane potential is set to its hyperpolarization potential, 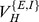. Additionally for principal cells, the calcium concentration is incremented by 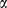, tuned such that 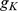 increases by 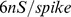. The cell remains in this hyperpolarized state for a refractory period (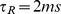), after which updates of the cell membrane potential are resumed (as per Equation 1).
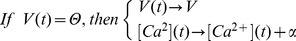
(5)


The default cell body and synaptic parameters [43] and noise parameters [42] were used throughout these simulations unless otherwise indicated, which may be found in Tables 2-3. The time constant of the excitatory feed-forward synaptic conductance, 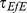 was set to 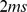 in line with a CT learning mechanism as explored in previous work [25].

### Lateral Connectivity

The input layer of the network incorporated a ‘Mexican hat’ lateral connectivity structure, featuring short-range excitatory connections and long-range inhibitory connections. To achieve this, the strength of connection between excitatory neurons within a layer becomes weaker with distance (while the strength of connections with inhibitory neurons remains constant). To set the excitatory spatial structure, the Euclidean distances between all principal (excitatory) neurons within a layer are calculated according to Equation 6.
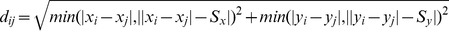
(6)


Here, 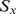 and 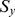 are the sizes of the 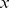 and 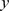 dimensions respectively, which together with the ‘min’ functions implement periodic boundary conditions, such that one-dimensional layers become circular (and two-dimensional layers become toroidal). All excitatory neurons within the input layer are then connected to every other excitatory neuron up to a radius of 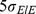 (given the probability of connection, 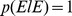). With the scaling factor 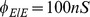, their synaptic weights (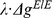) were set to a maximum of approximately 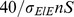, (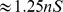 using the default value of 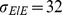) which then become exponentially weaker with increasing Euclidean distance, according to Equation 7.
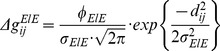
(7)


This accounts for the short-range excitatory component of the ‘Mexican hat’ weight profile, since the Gaussian weight profile means that more proximal principal cells share stronger connections than between more distal cells. The long-range inhibitory component is modelled by the uniform strength, full-connectivity between the principal cells and the inhibitory interneurons, which will come to dominate between more distal neurons as the excitatory connections become smaller.

The output layer featured the same connectivity between principal cells and inhibitory interneurons but had no lateral connections between the excitatory cells (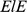) and hence no ‘Mexican hat’ synaptic weight profile.

### Synaptic Learning

To investigate the input dynamics upon learning, the Excitatory-Excitatory feed-forward connections between the layers were modified by an online, multiplicative form [44], [45] of Spike-Time-Dependent Plasticity formulated by [41] and described in Equations 8-10. Only the excitatory feed-forward connections (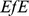) were modified through learning according to these rules, while all lateral connections (namely 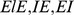) were fixed throughout each simulation.

Each synapse has a differential equation describing a plasticity variable 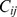 modelling a trace of recent presynaptic activity which may be thought of as the concentration of glutamate released into the synaptic cleft [41]. It is bounded by 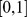 for 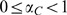 and is described in Equation 8, where 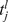 is the time of the 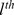 spike emitted by the 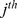 presynaptic cell.
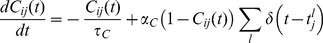
(8)


The presynaptic spikes drive 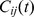 up at a synapse according to the model parameter 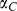 and the current value of 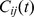 which then decays back to 0 over a time course governed by 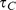.

The recent postsynaptic activity, 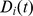, is modelled by Equation 9, which may be interpreted as the proportion of unblocked NMDA receptors as a result of recent depolarization through back-propagated action potentials [41]. Here 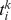 is the time of the 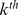 spike emitted by the 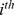 postsynaptic cell.
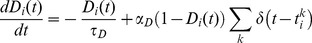
(9)


Based upon the instantaneous values of the plasticity variables 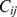 and 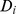, the strength of each feed-forward synaptic weight, 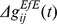, is then modified according to Equation 10 and governed by the time course variable 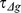.
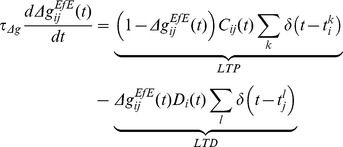
(10)


Note that the *post*synaptic spike train (indexed by 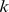) is now associated with the *pre*synaptic state variable (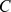) and vice versa. If 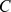 is high (due to recent presynaptic spikes) at the time of a postsynaptic spike, then the synaptic weight is increased (LTP). Conversely, if 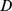 is high (from recent postsynaptic spikes) at the time of a presynaptic spike, then the weight is decreased (LTD).

Throughout the simulations presented, the default parameter values shown in Table 3 were used for the STDP model [41], except when they were systematically varied (as indicated) to assess their effect upon network performance.

The system of differential equations describing the dynamics of the cell bodies, synaptic conductances and synaptic plasticity are discretized with a Forward Euler numerical scheme and simulated with a numerical time-step 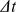 of 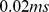. The code for this model has been made publicly available on our laboratory server (https://mac0.cns.ox.ac.uk/svn/SpikeNet/) and is also available upon request.

### Stimuli and Training

The stimuli used throughout these studies were abstract, homogeneous patches, represented by injecting tonic current into spatially separate pools of input-layer neurons. Contiguous blocks of neurons within these pools were gradually shifted across the input layer to represent successive overlapping transforms (translations) of each stimulus which which may be associated together in the output layer by Continuous Transformation learning [14]. In the initial simulation with two stimuli, a stimulus consisted of 64 neurons which was presented in 13 locations (transforms), with a shift of 16 neurons between each adjacent transform. This yields an overlap of 75% between contiguous transforms for the facilitation of the CT learning mechanism (as described in Figure 1).

During testing phases, all stimuli were presented individually to measure how the network responded specifically to each stimulus. However during training, the stimuli were presented together, such that the network never learnt about them in isolation. The untrained network was first tested in a ‘pretraining’ phase by presenting all transforms of all stimuli sequentially, each for a cue period of 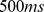. This phase therefore provides a baseline ‘pre-training’ behaviour to contrast with ‘post-training’ behaviour, in order to reveal the effects of learning in the feed-forward synapses.

**Figure 2:**
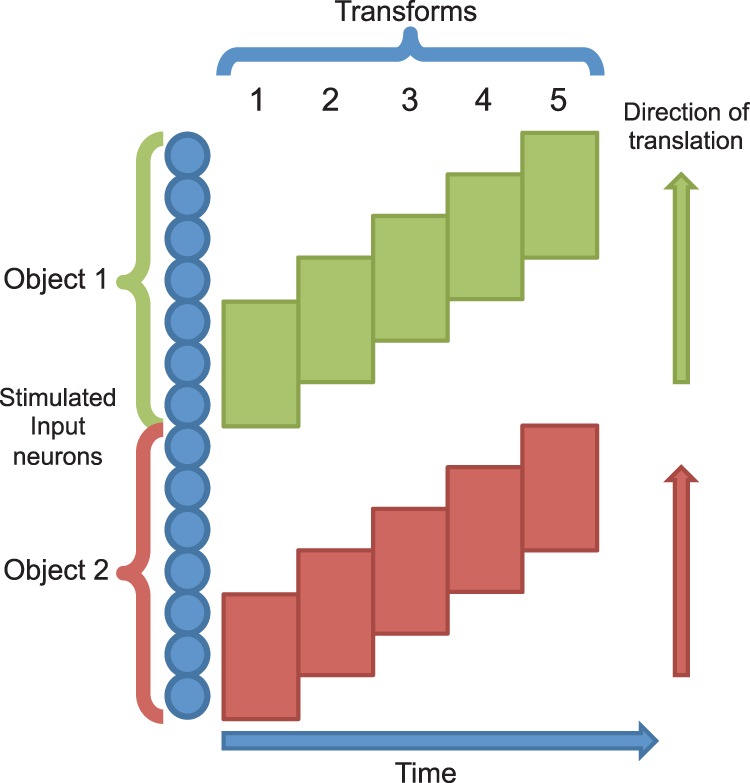
Training stimuli schematic. Two stimuli are presented simultaneously to the network by injecting current into neurons in separate parts of the input layer. These stimuli are then shifted with the same velocity across their respective parts of the input layer.

After saving the network outputs and resetting the dynamic variables for each cell and synapse, the training phase began where all transforms of all stimuli were presented for 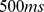 per transform. For combinations of stimuli, the direction of shift between transforms was randomly chosen but with each stimulus in the presented pair shifting in the same direction at the same rate (lock-step) to prevent any (slow) disentanglement through independent motion [17]. The presentation of all transforms of the pairs of stimuli constituted one epoch of training, and the training phase consisted of ten epochs in total. Figure 2 illustrates the multi-stimulus presentation paradigm with a simple example of two stimuli with five transforms each, over one epoch of training.

Once the network had been trained and the dynamic variables (except synaptic weights) reset, the ‘post-training’ testing phase was simulated in an identical way to the pretraining testing phase. The final outputs were then saved and analysed with the information theory algorithms described below.

### Network Performance Measures

The network performance is primarily assessed using two measures derived from information theory [46], [47], which reflect how well cells respond invariantly to a particular stimulus over several transforms but differently to other stimuli [48]–[50]. In so doing, these analyses measure the extent to which a cell possesses both *specificity* to the identity of a particular object (ideally by responding to one stimulus only) and *generality* to natural variations in its appearance (ideally by responding to all transforms of that stimulus) – the computational crux of visual object recognition [24].

While spiking dynamics are critical for how the network organises the stimulus representations, analysis of macaque visual cortical neuron responses has revealed that the majority of information about stimulus identity is contained within the *firing rates* rather than the detailed timing of spikes [5]. Accordingly, the network self-organizes through spiking dynamics but the information content (with respect to stimulus identity) is assessed through the output cell’s firing rates.

To measure the information conveyed by the responses of the output neurons, each transform of each stimulus was presented to the input layer of the network individually during a testing phase. Each neuron was allowed to settle after presentation of each transform, such that the activity due to one transform did not affect the responses to later transforms. The spikes of each output neuron were binned individually for each transform of each stimulus and the corresponding firing rate for each cell was calculated. Each cell’s responses were then used to construct conditional 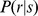 and unconditional 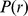 firing rate distributions. From these distributions, the stimulus-specific single-cell information, 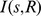, was calculated according to Equation 11. This measure quantifies the information conveyed by a particular cell through its complete set of responses to every transform of every stimulus, 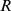, about a specific stimulus, 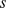.
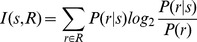
(11)


Good performance for a cell is indicated by a high (or maximal) information score, which would entail stimulus specificity, with generality across most (or all) transforms of that stimulus. In terms of the original firing rates, this would mean a large response to one stimulus regardless of its position (transform) and small responses to transforms of other stimuli. Such a cell may transmit relatively little information about other, non-preferred stimuli (for example, by responding indiscriminately to a number of other stimuli or unevenly to their transforms) but will still be very useful if it conveys maximum information for one particular stimulus. We therefore compute the maximum amount of information a neuron conveys about *any* of the stimuli rather than the average amount it conveys about the whole set of stimuli, 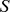 (which would be the mutual information).

If all the output cells learnt to respond to the same stimulus then there would be no discriminability and the information about the set of stimuli (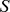) would be poor. To test this, the multiple cell information measure is used which calculates the information about the set of stimuli from a population of up to 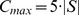 output neurons. This population consisted of the subset of up to five cells which had, according to the single cell measure, the most information about each of the two stimuli.

Ideally, we would calculate the mutual information – the average amount of information about which stimulus was shown from the responses of all cells after a single presentation of a stimulus, averaged across all stimuli. However, the high dimensionality of the neural response space and the limited sampling of these distributions are prohibitive to such an approach. Instead, a decoding procedure is used to estimate the stimulus 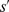 that gave rise to the particular firing rate response vector on each trial, as detailed below. Knowing (*a priori*) which stimuli have been presented, a probability table (confusion matrix) may be constructed (in the much lower dimensional space) between the real stimuli 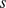 and the decoded stimuli 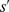, from which the mutual information is then calculated (Equation 12).
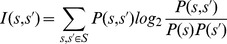
(12)


In this work, a Bayesian decoding procedure is used to infer the presented stimulus from the neural responses. For each cell in the ensemble vector, its firing rate response to each unknown transform is separately fitted to a Gaussian distribution of firing rates to each stimulus. Each stimulus-conditional distribution is parameterized by the mean and standard deviation of the cell’s sets of responses to transforms of each particular stimulus. Importantly, the unknown response is excluded from these parameterizations, hence a jack-knife cross-validation procedure is incorporated in the decoding process. This unknown response is then decoded by comparing it to each stimulus-conditional firing rate distribution to calculate from which it was most likely to have come, and so yield an estimate of 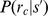. Taking the product of these probabilities over all cells in the response vector (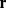) with 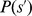 and then normalizing the resultant joint probability distribution gives an estimate of 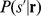 [51].

The calculated mutual information values were then corrected to compensate for the upward bias due to finite sampling [52]. As in previous work, only the first term of an analytically derived series was used, since this has been shown to be a good approximation [53], [54]. To smooth out the effects of random sampling for the neural ensemble, the information values were averaged over 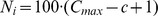 iterations, decreasing linearly (in this case from 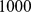 to 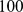) as the ensemble size, 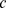, increases. The smoothed values were then clipped at the theoretical information limits to remove any artefacts caused by the approximate correction terms, before factoring them into the probability tables, 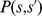. From these decoding, cross-validation and correction procedures, more reliable estimates of the true probabilities are obtained for calculating the multiple cell information measure [48].

This multiple cell information measure should increase up to the theoretical maximum 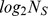 bits, (where 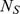 is the number of stimuli), as a larger population of cells is used, only if those cells have become tuned to different stimuli. A high information score from the multiple cell measure therefore indicates that all stimuli are represented in the ensemble of output cells, meaning that the network has good discriminability.

To assess the network performance across a range of parameter values, an ‘information score’, 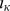 was calculated from the single-cell information described in Equation 11. For each stimulus, 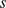, the number of cells which conveyed at least 95% (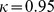) of the theoretical maximum information (in this case 0.95 bits) according to the single-cell measure was counted. The minimum number of such cells for any stimulus in the set was then found and normalised to a proportion of the total number of output cells. This ‘information score’ therefore expresses the information conveyed by the network about all transforms of the least well represented stimulus (see Equation 13).
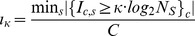
(13)


Here, 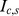 is the amount of information conveyed by a particular output cell, 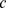, about a particular stimulus, 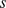 according to the single-cell information measure, 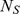 is the number of stimuli and 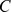 is the total size of (number of cells in) the output layer. Although this measure is derived from the single-cell information measure, taking the minimum proportion of cells across all stimuli means that non-zero values of 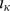 indicate that all stimuli are represented, fulfilling the role of the multiple-cell information analysis.

## Results

The results section first demonstrates the input layer dynamics and the ability of feed-forward plastic connections to take advantage of them in order to form independent, translation-invariant representations of each stimulus. These results are then further investigated by exploring their robustness to variations in key parameters. The stimuli are then expanded to a larger set of four (simultaneously presented during training) to show how the same mechanism may be applied in more ecologically valid scenes composed of more stimuli.

### Segmentation of Stimuli through Synchronisation

In the first simulation presented, a network was built with two layers of excitatory neurons (each with a separate pool of inhibitory neurons) as described in Equations 1-5 with parameters specified in Tables 1, 2, 3. The lateral connectivity was specified (between excitatory neurons within each layer only) as described in the methods section. Two stimuli were presented simultaneously to the network during training but individually during testing. This was simulated by injecting a current of 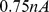 into the cell bodies of the sets of input neurons representing the particular transform (of a particular stimulus) during the presentation period of 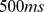 (either training or testing).

**Figure 3:**
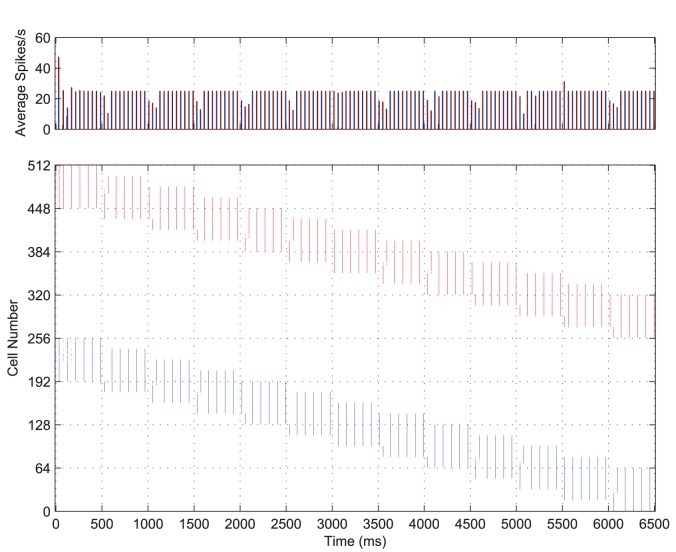
Input layer dynamics. The post-stimulus time histogram of global activity in the input layer (top) with the spike raster of the input layer (bottom) for the simultaneous presentation of each stimulus over all transforms, coloured according to stimulus. The first stimulus (shown in blue) consists of 64 neurons and its transforms are represented by the first (contiguous) half of the input layer (neurons 1-256) over which it gradually moves, while transforms of the second stimulus (shown in red) occupy the second half of the input layer (neurons 257-512). It can be seen from both the PSTH and raster plots that there is synchronisation of spikes within each stimulus representation and desynchronisation of spikes between them.

While both stimuli are represented simultaneously and with equal strength, the input layer neurons rapidly adjust the timing of spikes such that each stimulus is represented separately to the other through time. That is, the constellation of features representing a stimulus are synchronised with respect to one another and desynchronised with respect to the features of the other stimulus (see Figure 3, bottom). Throughout the course of stimulation, these two competing representations alternate (as shown in the PSTH, Figure 3, top), each with a regular frequency of approximately 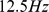. This means that, for both stimuli combined, the input layer exhibits 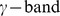 oscillations at slightly less than 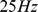.

**Figure 4:**
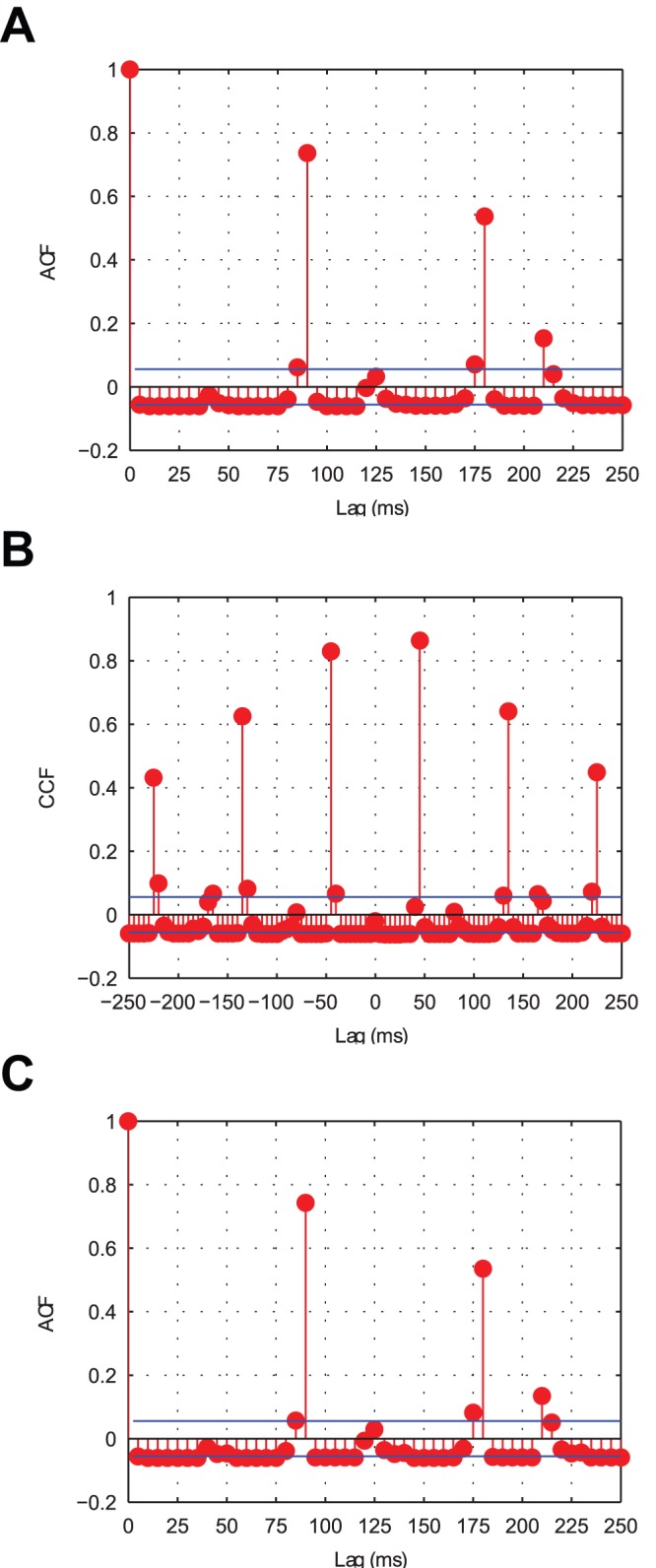
Input layer correlation functions. After binning the input layer spiking activity (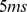 bins) the auto-correlations were plotted for Stimulus 1 (**A**) and Stimulus 2 (**C**) in addition to the cross-correlation between the stimuli (**B**). Both stimuli exhibit positive auto-correlations approximately every 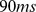, indicating that this is the period with which each is repeated. The cross-correlation shows strong peaks approximately every 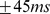, suggesting that the representations of the two stimuli are interleaved through time in anti-phase oscillations.

Looking at the combined PSTH (Figure 3) it is clear that the two competing populations of input neurons representing each stimulus have pushed one another out of phase, as the volleys of spikes (and frequency bars) for each stimulus are interleaved through time. This is confirmed by the cross-correlation (Figure 4, **B**) which shows that the volleys representing the two stimuli are positively correlated with lags of approximately 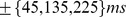 and anti-correlated elsewhere meaning that they are separated by a period, 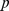, of approximately 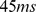 (with the positive cross-correlations corresponding to 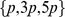). Furthermore, the auto-correlations for each stimulus’ populations of input cells (Figure 4, **A** and **C**) show that the volleys are repeating through time approximately every 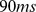 (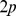).

To understand this phenomenon, consider the features (excitatory input neurons) representing a particular stimulus. For both populations (representing each stimulus), the external stimulation is identical in terms of time course and amplitude. This causes the neurons representing both stimuli to fire together initially, as can be seen in the first 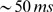 of Figure 3. However, the noise in the neurons’ cell potentials means that one population (or subpopulation) will by chance, quickly come to dominate the initial competition. These cells transmit action potentials to their neighbouring cells, thus raising the cell potentials of those nearby neurons and encouraging neurons which represent features of the same object to also fire.

Compared to excitatory neurons representing other features of the same object, those representing the second object are spatially much further away. As such, they do not receive as much excitation through the lateral connections, which exponentially decrease in strength with distance (see Equation 6). Instead, they receive a wave of inhibition from the mutually connected inhibitory interneuron population, which suppresses their firing. Since the principal cells representing the first object have now fired, they will have self-inhibited through their adaptation mechanism, making it relatively harder for them to fire soon after. Neurons representing the first stimulus are therefore less able to compete with the second population of input neurons (through the inhibitory interneurons), which are then able to fire their own synchronised spike volleys. The second population of cells then temporarily suppresses the first population by the same interneuron-mediated interaction, until they too self-inhibit through adaptation and the cycle repeats.

### Learning Translation-invariant Representations

The process described for the formation of anti-phase input representations, when coupled with CT learning [25] in the feed-forward plastic weights, is shown here to lead to the formation of translation-invariant representations in the output layer. If Spike-Time-Dependent Plasticity (STDP) is used, the output neurons will be selective in terms of which population of input neurons they associate with, as each stimulus representation is separated through time. This process persists as the objects translate across the input layer until all transforms of a particular stimulus are potentiated onto a distinct set of output neurons.

**Figure 5:**
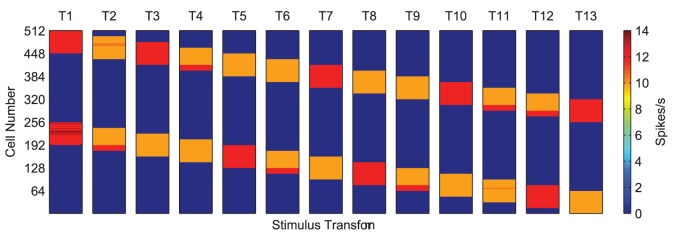
Firing rate plots of the compound training stimulus across all transforms. Each transform of the two combined stimuli (labelled 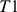 to 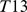) are plotted showing the two input representations move in lock-step across the input layer. On the basis of the input neurons' firing rates alone (indicated by the colour of heat map in Spikes/s) it is very difficult for the next layer of neurons to distinguish between the transforms of each of the testing stimuli when presented together.

In contrast to the precise timings of the action potentials shown in Figure 3, it would be very difficult for the output layer to distinguish between the two input stimuli on the basis of firing rates alone (given the full excitatory feed-forward connectivity between the layers). The 13 transforms of the ‘compound’ training stimulus are plotted as a rate-coded representation in Figure 5 for comparison.

**Figure 6:**
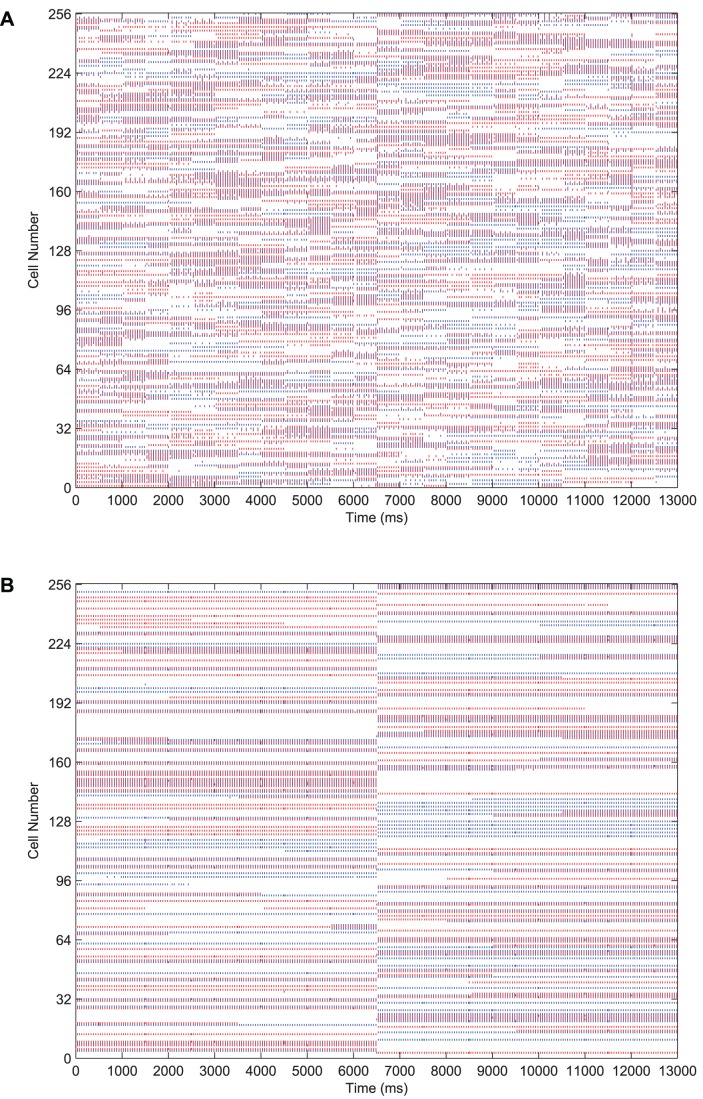
The effect of training upon the output layer. Raster plots for all output layer neurons during presentation of each transform of each stimulus are shown before and after training. Before training (**A**), the output cells respond randomly to transforms of each stimulus. After training (**B**), the majority of output cells have become object selective and translation-invariant.

In Figure 6 we present results demonstrating the formation of cells in the output layer which are selective to one of the two stimuli presented to the network during training, yet invariant to most or all transforms of that particular stimulus. For the testing phase shown in these raster plots, each transform of Stimulus 1 is presented in sequence for 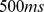 each, followed by the sequence of transforms for Stimulus 2, each for the same duration. In the case of the trained network, this leads to a shift after 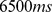 where a separate population of output neurons become active to represent the transforms of the second stimulus.

**Figure 7:**
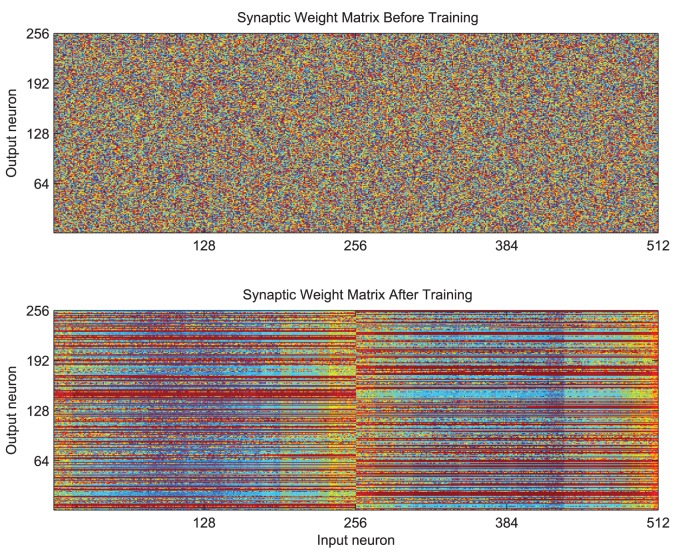
The effect of training upon the organisation of the feed-forward synaptic efficacies. The strength of the synaptic weights are indicated by the colour (red being high and blue being low). Before training (top) the weight matrix is random (unstructured). After training (bottom) there are strong connections from all the inputs representing all transforms of one stimulus to particular output neurons (indicated by horizontal red stripes) and likewise for the input neurons representing transforms of the other stimulus to a different set of output neurons.

The effect of training is also clear from the structure in the weight matrix of feed-forward excitatory synaptic conductance strengths as shown in Figure 7. These synaptic weights are initialised to random values drawn from a uniform distribution (top) but attain a clear structure through the process of training (bottom). After training, particular output neurons (shown on the 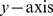) can be seen to have striations of large weight values extending across, for example, the first half of the input layer (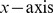) corresponding to all transforms of the first stimulus. In contrast, neurons in the second half of the input layer have formed strong feed-forward synapses with other output neurons, which have come to represent all transforms of the second stimulus.

**Figure 8:**
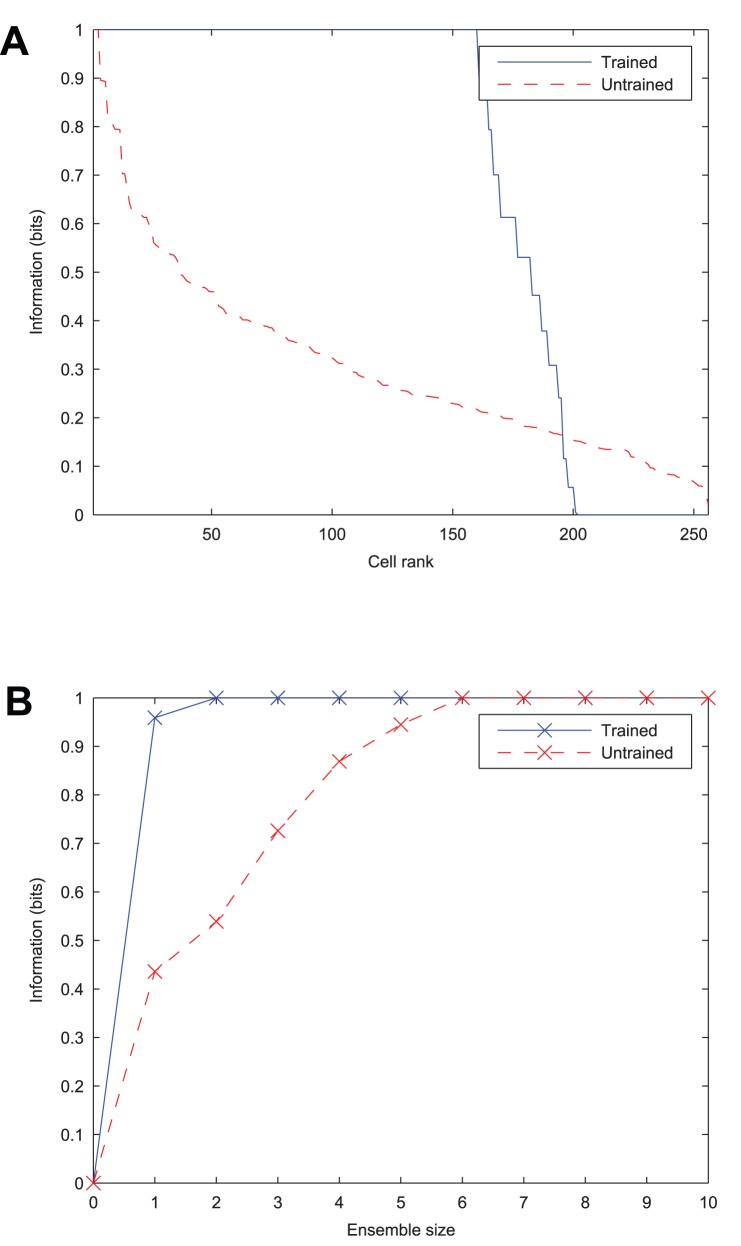
The effect of training upon the information content of the output layer. Before training, both information measures can be seen to be low. After training, the single cell information measure (**A**) shows that most of the cells in the output layer are maximally informative in discriminating between the stimuli and the multiple cell information measure (**B**) shows that both stimuli's transforms have been learnt by the network.

As a quantitative measure of the network's ability to learn to form transformation-invariant representations of each stimulus, information analysis plots of the output layer are given in Figure 8. The plot of the single-cell information measure shows that a large proportion of output cells transmit the maximum information (1 bit) across all transforms of the stimuli, meaning that they unambiguously signal which stimulus is being presented. The multiple-cell information measure plot shows that both stimuli are represented in the output layer, as it also attains the maximum information level.

### Spread of Lateral Excitatory Connections

During preliminary explorations of the parameter space, earlier simulations revealed the gradient in the strength of lateral excitatory connections to be a crucial element in generating anti-phase relationships in inputs presented to the network. Further investigation was needed to understand how the spread of such connections relative to the size of the stimuli relates to the network's ability to learn stimulus-specific translation-invariant representations.

If the radius of excitation grows too large relative to the spacing between the stimuli, then two stimuli are encouraged to fire together, leaving the postsynaptic neurons unable to distinguish between their synchronised activity. If the radius becomes too small relative to the size of the stimuli, the input representation becomes fragmented as not all features of the object are synchronised by the lateral excitatory connections and only partial views (or a subset of transforms) are learnt about by postsynaptic neurons. In summary, for large 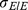 specificity was found to suffer, whereas for small 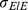 invariance learning suffers.

**Figure 9:**
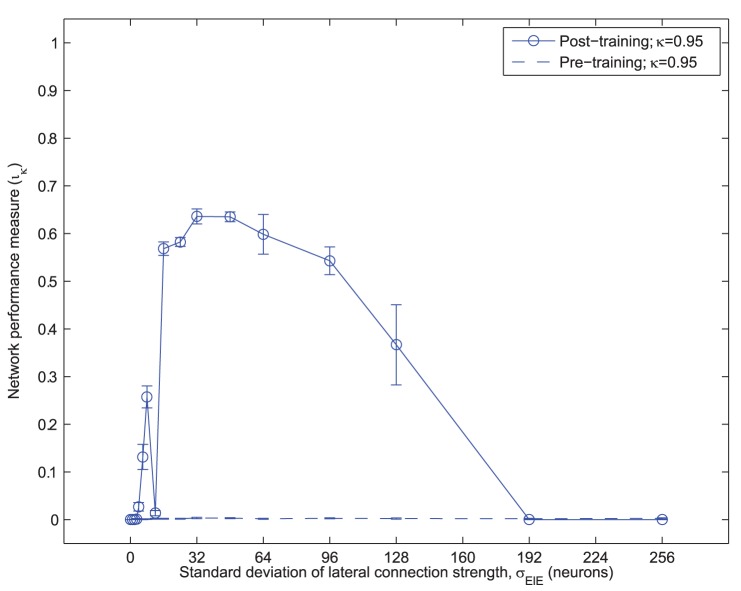
Network performance versus lateral connection spread. The mean network performance measure (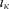) is plotted against the standard deviation of the lateral excitatory connection strength (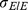) for ten random seeds, with the standard error of the mean indicated by the whiskers. It can be seen that network performance is robust to approximately a four-fold increase in 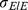.

Here we used the parameters from the optimal simulation (Table 1, with 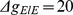) and varied 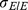 from 0 to 256. The results of running ten different random seeds are shown in Figure 9, with the mean information score, 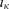, plotted as points and the standard error of the mean over the ten random seeds plotted as whiskers.

From this analysis, the optimal standard deviation of the lateral excitatory connection profile was found to be 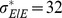, (1/2 the width of each stimulus and 1/8 of the average inter-stimulus distance). Network performance (as measured by the information score) was also found to be more tolerant to larger spreads of the weight profile than to smaller values of 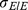, as shown by the relatively steep decline on the left side of Figure 9. The optimal value was used in subsequent simulations throughout this paper.

### Temporal Specificity in Learning

In the simulations presented so far, the alternation of input representations on successive gamma cycles allows the output layer to exploit learning through Spike-Time-Dependent Plasticity (STDP) – a temporally sensitive learning rule. It is hypothesised that if the form of STDP is made less specific (such that it begins to resemble a firing-rate based learning rule) then the advantage of the self-organised perceptual cycles in the inputs will be lost, as the learning rule will no longer be specific enough for a given population of output cells to learn about a particular input without interference from the other. In other words, the alternation of the input representations will effectively be too fast for output cells to learn about one or the other discriminatingly. In this case, the different input representations will be associated onto the same output cells.

**Figure 10:**
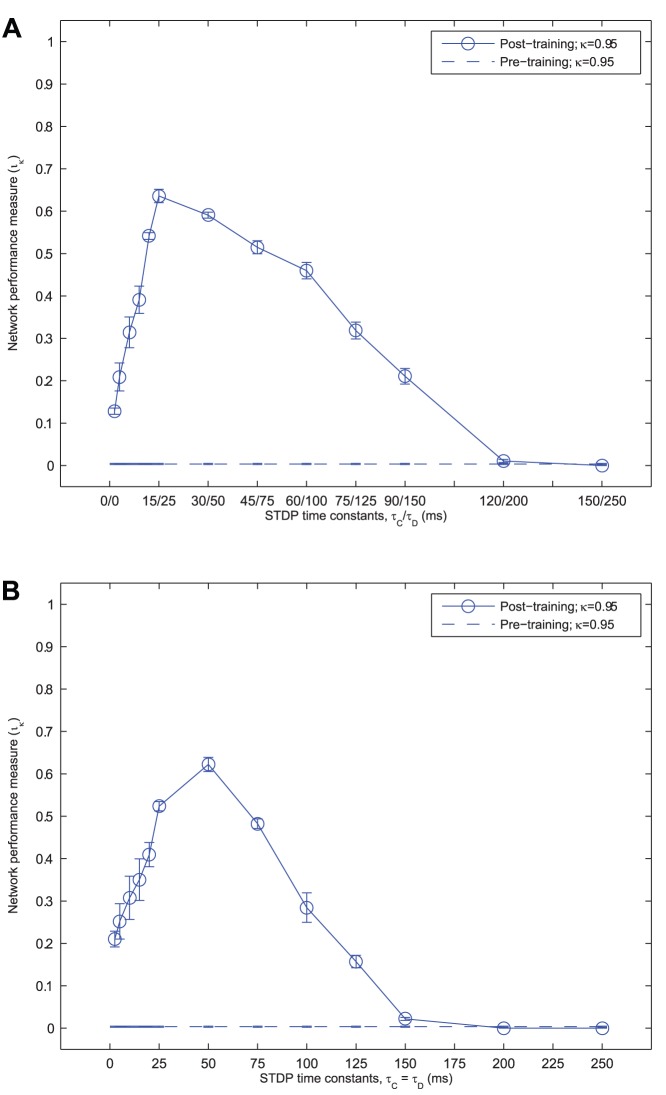
Network performance versus STDP time constants. The mean network performance measure (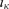) is plotted against the STDP time constants (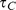 and 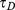), maintaining a constant asymmetric ratio, 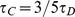 (**A**) or symmetric learning windows, 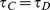 (**B**). The standard error of the mean across the ten random seeds for each set of simulations is also indicated by the whiskers around each mean value. In each case, the network performance is shown to be robust to a large span of STDP time-constants. Lengthening the time constants eventually reduces performance to 0 due to association across stimuli, whereas performance remains reasonably high for very short time constants, around 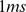.

To test this, further simulations were run with a range of STDP time constants (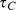 and 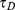), both symmetrical (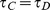) and asymmetrical (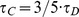). To summarise the network performance, the information score, 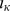 was calculated as before according to Equation 13. The results of these simulations are presented in Figure 10 (**A**) for asymmetrical time constants (with a larger LTD time window) and Figure 10 (**B**) for symmetrical time constants.

As the STDP time constants deviate from the default values, 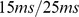, the network performance can be seen to deteriorate (although with symmetrical learning windows, the optimal time constant was found to be longer at 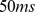). When the learning time constants are shortened, network performance is reduced since only partial fragments of the stimuli are synchronised within the more restrictive time window. Consequently, associating all transforms of a particular stimulus together becomes a more difficult task. By lengthening these time constants, the network performance also decreases (although more gradually) as the oscillations of both stimuli start to experience both LTP and LTD together.

From inspecting the output layer cell response properties (not shown) for simulations with STDP time constants of 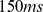 or longer, it was confirmed as hypothesised that the learning rule is no longer temporally specific enough for separate sets of output cells to learn about each stimulus independently. Instead, one set of output cells tend to form which are invariant to all transforms of both stimuli.

### Lateral Connections

In order for features of the same object to generate synchronised firing in the input cells which represent them and yet desynchronise the firing between more distant populations of cells representing different objects, a gradient of lateral excitation is necessary. The consequence of this architecture is that cells which are close together are more mutually supportive than those which are further apart.

To test this, the same simulation was rerun under two different conditions; firstly with no lateral excitatory connections in the input layer and secondly, keeping the lateral excitatory connections but flattening the Gaussian profile of their strength (i.e. all lateral excitatory connections were of the same efficacy irrespective of the distance between the neurons). In each case, the strength of inhibition was adjusted to prevent saturated firing through positive feedback.

**Figure 11:**
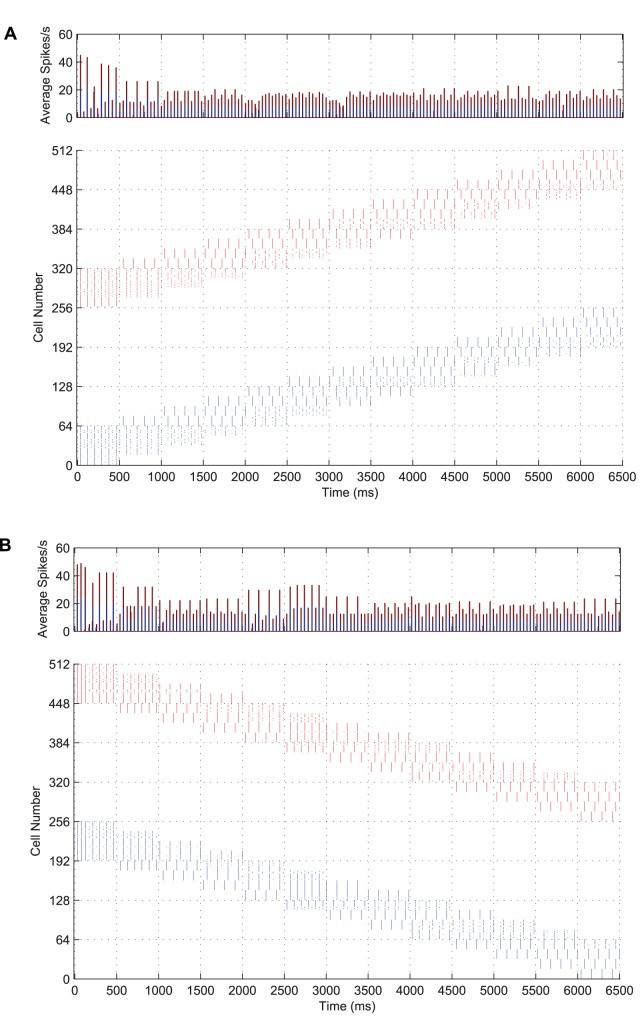
Input layer activity without lateral excitatory connections and with no strength gradient. Both post-stimulus time histograms (top row) and input spike rasters (bottom row) are shown for a network with no lateral excitatory connections (**A**) and a network with a flat synaptic efficacy profile in all its lateral connections (**B**). In each case, the spike volleys representing each stimulus are disorganised, with no global synchronisation within a stimulus and no desynchronisation between stimuli.

In Figure 11 we present input rasters from a typical simulation with no lateral excitatory connections in the input layer (**A**) and one with a flat strength profile and retuned lateral connection strength (**B**). It is evident in each plot that, not only have the perceptual cycles between the input stimuli disappeared, but also the coherence of the representations, shown by the limited local synchronisation between subsets of features within each stimulus.

Consequently, with no temporal structure in the spike timings of cells representing the transforms of the input stimuli, the output layer does not manage to discriminate between the two inputs and hence the single and multiple cell information measures (not shown) are no better than the untrained network (essentially no improvement on random feed-forward connectivity). This suggests that a distance-based gradient in the lateral connection strength profile is a necessary element of this model for forming perceptual cycles in the input layer and that these are the basis for learning separate object representations in the output layer.

### Cell Firing Rate Adaptation

**Figure 12:**
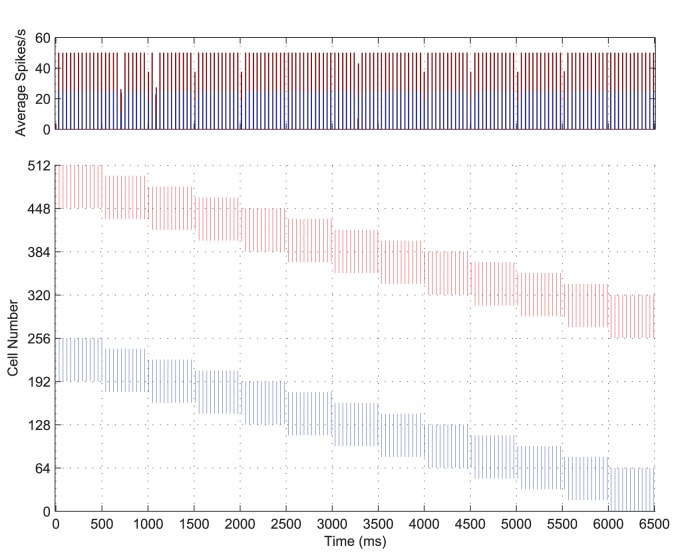
Input layer activity with no cell firing-rate adaptation. Post-stimulus time histogram of global activity in the input layer coloured according to stimulus (top) with the spike raster of the input layer (bottom) for the simultaneous presentation of each stimulus over all transforms. Very quickly, both populations of input neurons start firing together and remain synchronised throughout the epoch.

Adaptation has been found to be a necessary element in generating anti-phase representations between input stimuli, also known as ‘perceptual cycles’ [22]. Without cell firing-rate adaptation, the entire population of excitatory input cells was synchronised by the action of the inhibitory interneurons. This can be seen in Figure 12, which may be contrasted with the input layer raster plot of Figure 3 showing both stimuli being represented in anti-phase cycles. Without a mechanism of self-inhibition and the effects of cell membrane noise and random initialisation to help randomly select an initial winner to begin the oscillations, the two populations would continue to fire as one. As such, it was much harder to train the network to form separate output representations of each stimulus, as without the dynamic of perceptual cycles, both stimuli would typically be associated onto the same output neurons.

This was also found to be the case for a wide range of parameters, including the strength of the lateral connections, the standard deviation of the distribution of their strengths, (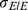), and the strength of excitatory to inhibitory connections and inhibitory to excitatory connections. Without the time-varying degree of competition (provided by the self-inhibiting effects of cell firing-rate adaptation in this case), the perceptual cycles can no longer be formed and so are not observed in the results.

### Capacity

While presenting pairs of stimuli to the network during training is an advance on presenting stimuli in isolation, there is still much scope for more biologically realistic and therefore improved ecological validity of the simulations. In the following simulations we aim to investigate the capacity of the network by presenting larger numbers of stimuli (four) simultaneously during training. The size of the network and the number of transforms remain the same but as the number of stimuli doubles, the size of the stimuli and the shift between transforms both halve to 32 and 8 neurons respectively. To encourage synchronisation within object representations and desynchronisation between object representations, the spread of excitatory lateral connections was reduced slightly (to 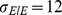) and the injected current was also reduced to allow for a slower frequency of firing (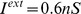).

**Figure 13:**
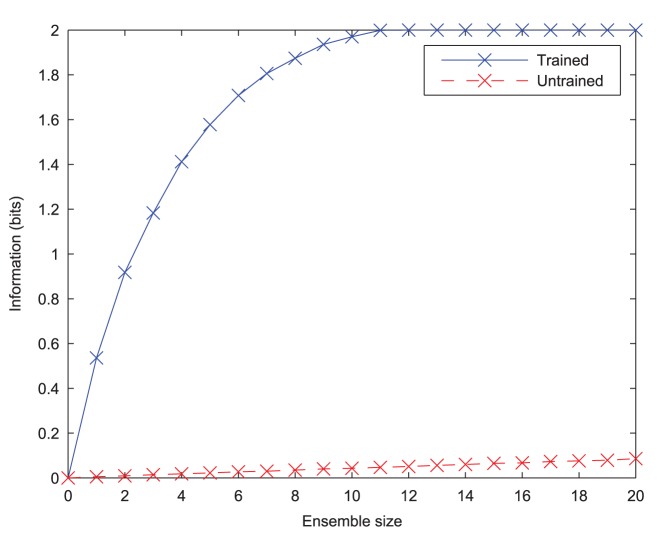
Input layer dynamics with four stimuli. Post-stimulus time histogram of activity in the input layer coloured according to stimulus (top) with the spike raster of the input layer (bottom) for the simultaneous presentation of four stimuli over all transforms. The first stimulus consists of 32 neurons and its transforms are represented by the first (contiguous) quarter of the input layer (neurons 1–128) over which it gradually moves, while transforms of the second, third and fourth stimuli occupy the subsequent quarters of the input layer. The four stimulus representations are generally internally synchronised and interleaved through time in perceptual cycles (for example, Transform 9: 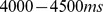). Occasionally, two of the stimulus representations become synchronised, however the stimulus combination is random and can be seen to change between different transforms.

Using the same simulation paradigm as described above (except for the changes necessary to accommodate four stimuli as discussed), the network was trained with all four stimuli presented simultaneously translating across their portions of the input layer. The PSTH and raster plot of the input layer with four stimuli are shown in Figure 13. It can be seen from these plots that the four populations of input neurons have organised themselves into internally synchronised volleys, which are out of phase with respect to the spikes from neurons representing the other three stimuli. This is qualitatively very similar to the case with two stimuli presented during training except that the volleys of spikes for a particular stimulus fire approximately once every three or four cycles (as opposed to every two cycles) and that there is occasionally some synchronisation between volleys representing transforms of different stimuli.

**Figure 14:**
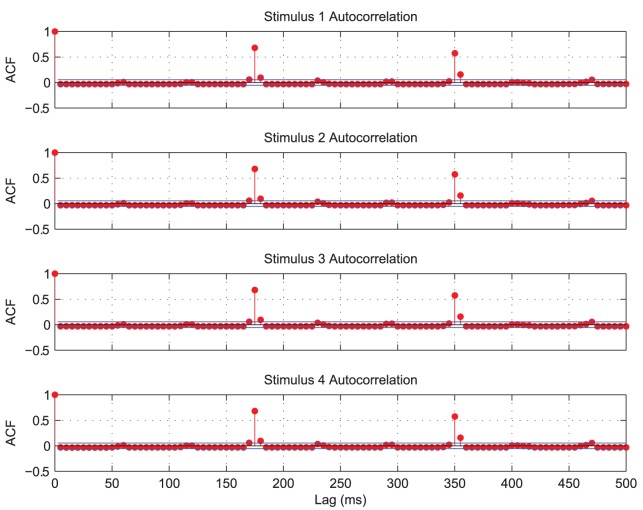
Input layer auto-correlation functions with four stimuli. The four populations of input neurons representing each stimulus were binned separately (in 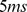 intervals) and auto-correlations were plotted for each population. The auto-correlations are found to be significant (when they rise above the blue line) approximately every 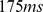, indicating a combined period of approximately 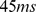 for an ideal separation of the four stimuli.

The auto-correlations for each of the four populations of input neurons representing each of the four stimuli are plotted in Figure 14. They each show a high correlation repeated approximately every 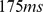, indicating the period of oscillation for each stimulus. For an ideal separation of the 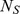 competing stimuli, the combined input representations (across all stimuli) should oscillate at approximately 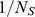 of the autocorrelation periods (the ideal separation period, 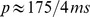). This implies that at least one population of input cells should fire approximately every 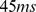 in a repeating cycle.

**Figure 15:**
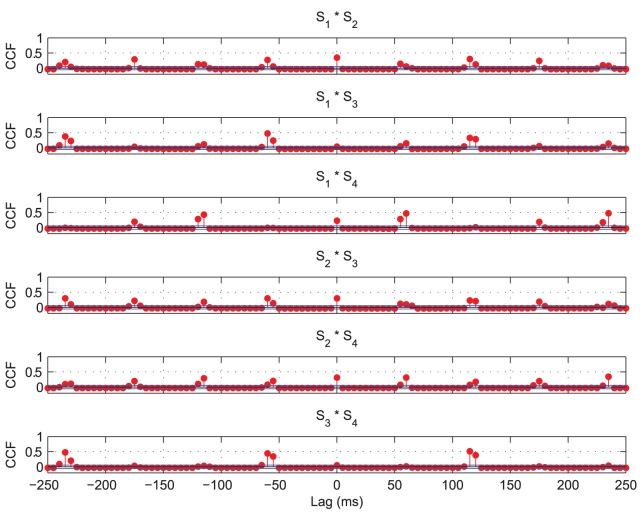
Input layer cross-correlation functions with four stimuli. Significant cross-correlations can be seen at multiples of approximately 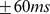, indicating that at least one stimulus is represented every 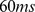. There are also significant cross-correlations at 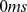 lag for some of the pairs of stimuli, showing that on this particular training epoch, the firing of those stimuli was still (at least partially) synchronised. However, inspecting the data from other training epochs confirmed that the synchronised pairs changed between presentations.

The cross-correlations of spiking activity during a single epoch of training are shown in Figure 15 for each of the six possible combinations of two stimuli. Significant cross-correlations are observed approximately every 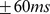, contrary to the expected peaks at lags of 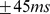 suggested by the period of the auto-correlations (Figure 14). This means that occasionally two of the stimulus representations tend to be synchronised, as evidenced by the 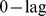 cross-correlations for some pairs of stimuli in Figure 15 and in the occasional coincident spiking activity of Figure 13.

**Figure 16:**
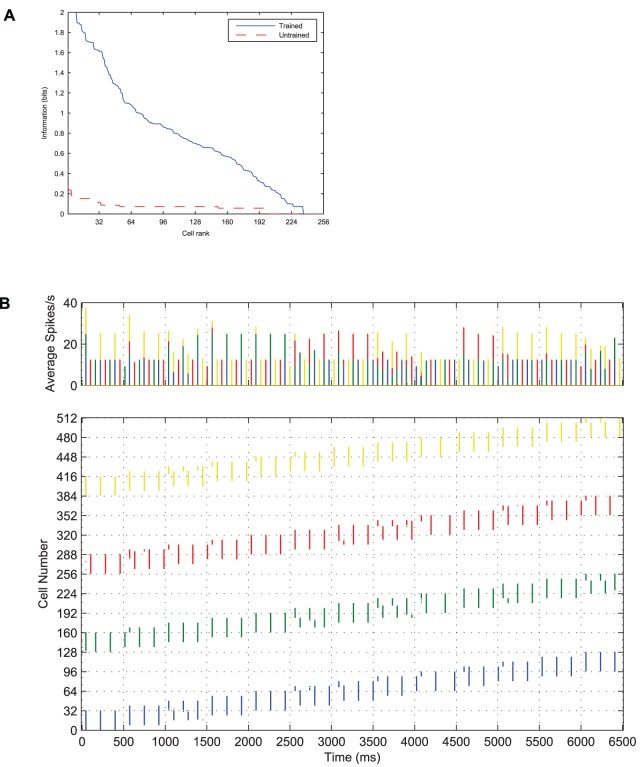
The effect of training with four stimuli upon the information content of the output layer. It can be seen that before training, the information content according to the single cell (**A**) and multiple cell (**B**) information measures is extremely low. After training the network, the single cell information measure shows that a number of cells in the output layer are maximally informative in discriminating between the stimuli. Similarly, the multiple cell information measure also reaches the maximum 2 bits, showing that all four stimuli's transforms have been learnt by the network.

Importantly, the coincident representations tend to occur randomly between different combinations of transforms at different times (as shown in Figure 13) and on different training epochs, as was confirmed by examination of rasters and cross-correlations for the other training epochs (not shown). With this lack of consistency between coincident stimulus representations and a sufficient degree of training (extended to 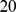 epochs for these results), cells in the output layer were eventually able to learn *independent* representations of each stimulus, as confirmed by the information analysis of Figure 16.

The Information plots for four stimuli (Figure 16) show that a number of neurons in the output layer have been trained to convey the theoretical maximum amount of single-cell information possible which has risen to *two* bits (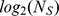, where the number of stimuli, 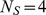), rather than one bit in the case of two stimuli. This means that these cells have been able to learn to unambiguously signal which stimulus is being presented from any of its transforms, despite always experiencing all four stimuli together during training, and the lack of perfect stimulus separation. Furthermore the cells in the output layer can collectively identify each of the four stimuli across all transforms as indicated by the multiple cell information measure reaching the theoretical maximum of two bits.

## Discussion

This paper has investigated how a model of the primate ventral visual system may develop neurons which are selective to a particular *individual* stimulus and respond invariantly to it over a set of learned transforms, even though the network is only exposed to visual scenes containing *multiple* stimuli moving together. Previous work has investigated this issue using rate-coded neural networks whereby the precise times of action potentials are not simulated explicitly but replaced by a temporal average. To overcome the ‘superposition catastrophe’ of associating the simultaneously presented stimuli together onto the same output neurons through a rate-based Hebbian learning rule, these earlier studies had to present the stimuli in different combinations on different learning trials [15], [16], or the stimuli had to be shown moving independently of each other during training [17]. The work presented in this paper has demonstrated how by using a spiking neural network, (in which the times of the action potentials are explicitly modelled) output neurons can learn separate representations of visual stimuli which are always seen moving together in lock-step during training but which are separated in space.

Importantly, this model incorporates ‘Mexican hat’ lateral excitatory connectivity within the input layer with cell firing-rate adaptation. The effect is that the short-range (exponentially declining) excitatory lateral connections help to synchronise localised clusters of input neurons (representing a stimulus). Conversely, the long-range (global) inhibition and firing-rate adaptation constitute a delayed self-inhibition mechanism, pushing the firing of one cluster out of phase with respect to the other, so causing them to oscillate through time. The first simulations demonstrate the resulting effect, which is to synchronise the firing of neurons representing one particular stimulus and desynchronise them with respect to the ensemble representing the other stimulus.

The alternating representations in the input layer, arising from the detailed properties of the network, were then shown to facilitate learning about the stimuli individually. When combined with the temporal specificity of STDP in the feed-forward excitatory connections, this temporal separation between the oscillating input representations allows for different pools of output neurons to learn about each stimulus separately. Furthermore, this input layer dynamic persists as the stimuli transform (translate across the input layer) such that the output neurons also build translation-invariant representations of the individual stimuli through the CT learning mechanism [14], [25].

In agreement with earlier work, the ability of the input layer to form the perceptual cycles of the individual stimuli (when presented simultaneously) was found to be be critically dependent upon a mechanism of delayed self-inhibition [22], [27] – in this case, cell firing-rate adaptation. The adaptation model used here is a more realistic implementation than in previous work [22], [55], yet instantiates the same core principle, thus indicating a convergence of views.

In addition to cell firing-rate adaptation, the presented simulations also demonstrated the importance of the lateral excitatory connectivity in generating perceptual cycles between the input representations. A ‘Mexican hat’ functional architecture is often taken to mean lateral connectivity with short-range excitation and long-range inhibition [35], [36]. This connectivity was modelled here through a gradual weakening of the excitatory lateral connections with increasing distance plus uniform strength, fully laterally connected inhibitory interneurons (representing a long-range or global inhibitory mechanism). By flattening the profile of these lateral excitatory connections or removing them altogether, the perceptual cycles were extinguished. This made the input representations disorganised and unable to facilitate translation-invariant learning of independent object representations in the feed-forward connections to the next layer.

The role of lateral connectivity was also explored by systematically varying the standard deviation of the lateral excitatory connection strength, 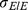, to assess its impact upon network performance. It was found that if this parameter was too small, then not all ‘features’ of a particular stimulus were synchronised – in other words, the lateral excitatory connections were unable to promote coherence of the stimuli (intra-stimulus synchronisation). Alternatively, with too large a spread of 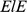 strength, the neurons representing features of both stimuli are encouraged to fire in phase with each other, abolishing the perceptual cycles found with intermediate values. At each extreme, the disruption caused to the input representations had a negative impact on network performance, meaning separate translation-invariant representations were less likely to form.

The problem of synchronising independent objects with a large radius of excitation may be alleviated with more realistic inputs and architecture. In V1, cells sensitive to a particular bar or edge orientation are laterally connected to other cells of similar orientation preference through excitatory synapses [57], providing a means of contour integration [35]. Similarly, excitatory lateral projections in V2 appear to be between cells with a wide range of orientation preferences but avoid orthogonal orientations [58]. If these ‘feature-aligned’ lateral connections are strong relative to the undirected ‘Mexican hat’ lateral connections, this architecture would allow distinct objects (with unaligned edges) to be closer together in the visual field, without their representations (undesirably) synchronising. Equivalently, the (‘Mexican hat’) radius of excitation could be larger without the collapse of meaningful perceptual cycles between distant, independent objects.

Conversely, if the edges of two stimuli were aligned (particularly if they are close together), their neural representations would tend to synchronise, eliminating the anti-phase relationship in their oscillations and therefore binding them as a single percept [38], [39], [59]–[61]. However, this would be advantageous if the ‘two’ stimuli were actually a single occluded object, suggesting a neurophysiological basis for the Gestalt ‘continuity principle’ and perceptual phenomena such as illusory contours [62]. If feature alignment is a major architectural principle of early visual areas, along with decreasing strength (or probability) of connection with increasing distance, the two features would work synergistically to both, segment proximal stimuli and integrate distal contours as appropriate. This would predict that the radius of excitatory lateral connections, especially between neurons representing aligned features, should be large relative to the distance spanned by the cortical representation of an object.

The ability of this model to learn output representations which are both selective to a particular stimulus and invariant across its transforms was found to be dependent upon a number of key properties. The temporal specificity of the STDP learning rule was explored through systematically varying the time constants of the LTP and LTD time windows. If the time constants were too short, the output neurons were unable to learn translation-invariant stimulus representations successfully as the learning rule became too sensitive to the timing jitter of the input spike volleys. Conversely, if the STDP time constants became too long, there was not enough temporal specificity to isolate the potentiation of a particular output neuron to just one stimulus and both stimuli associated onto the same output neuron.

The paradigm of translation-invariance learning employed here has been used as an example of more general forms of transformation-invariance learning, as used in several previous studies [5]–[7], [25], [63]–[67]. Invariance learning with other forms of transformation, such as scalings and rotations, should operate in a similar manner. Small changes in view of the same object (including rotations and scalings) are likely to activate an overlapping set of bar and edge detector neurons in V1 and other early visual areas. This would enable the CT learning mechanism to associate similar transforms together onto the same downstream neurons, as exemplified by the results presented here. Since this was not easy to demonstrate with the abstract stimulus representations used in these simulations, future work would benefit from validating this point with more realistic stimuli undergoing other forms of transformation.

In summary, this paper has shown one way in which spiking neural network dynamics may support mechanisms necessary to solve key problems in learning specificity to object identity and generality across object transforms. In particular, the simulations have shown how competitive oscillations and Spike-Time-Dependent Plasticity may be critical to enabling the primate ventral visual system to segment natural scenes composed of multiple stimuli, thereby forming independent and translation-invariant representations of each object in higher layers. As such, it indicates the importance of using detailed spiking models (over simpler rate-coded models) to more fully understand the learning processes involved in biological visual object recognition.
