# Correction: How Lateral Connections and Spiking Dynamics May Separate Multiple Objects Moving Together

**DOI:** 10.1371/annotation/7f6b65d9-a88d-4bdf-a174-48b1bc99b192

**Published:** 2013-08-07

**Authors:** Benjamin D. Evans, Simon M. Stringer

Figures 13 and 16B were accidentally switched. The image appearing as Figure 13 belongs with the title and legend of Figure 16 (which describe parts A and B of that figure), and the image appearing as Figure 16B belongs with the title and legend of Figure 13. 

